# Association of folic acid dosage with circulating unmetabolized folic acid in Chinese adults with H-type hypertension: a multicenter, double-blind, randomized controlled trial

**DOI:** 10.3389/fnut.2023.1191610

**Published:** 2023-09-14

**Authors:** Ping Chen, Linlin Tang, Yun Song, Binyan Wang, Xianhui Qin, Nan Zhang, Yaping Wei, Xiping Xu, Ziyi Zhou, Qiangqiang He, Lishun Liu, Sultan Mehmood Siddiqi, Xiao Huang, Xiaoshu Cheng, Genfu Tang, Yong Duan, Houqing Zhou, Jie Jiang, Sha Li

**Affiliations:** ^1^College of Pharmacy, Jinan University, Guangzhou, China; ^2^State Key Laboratory of Natural Medicines, Research Center of Biostatistics and Computational Pharmacy, China Pharmaceutical University, Nanjing, China; ^3^Institute of Biomedicine, Anhui Medical University, Hefei, China; ^4^National Clinical Research Center for Kidney Disease, State Key Laboratory for Organ Failure Research, Guangdong Provincial Key Laboratory of Renal Failure Research, Guangzhou Regenerative Medicine and Health Guangdong Laboratory, Division of Nephrology, Nanfang Hospital, Southern Medical University, Guangzhou, China; ^5^Key Laboratory of Precision Nutrition and Food Quality, Department of Nutrition and Health, College of Food Sciences and Nutritional Engineering, China Agricultural University, Beijing, China; ^6^Shenzhen Evergreen Medical Institute, Shenzhen, China; ^7^Graduate School at Shenzhen, Tsinghua University, Shenzhen, China; ^8^Department of Cardiology, The Second Affiliated Hospital of Nanchang University, Nanchang, China; ^9^School of Health Administration, Anhui Medical University, Hefei, China; ^10^Department of Clinical Laboratory, The First Affiliated Hospital of Kunming Medical University, Kunming, China; ^11^Department of Clinical Laboratory, Fuwai Hospital Chinese Academy of Medical Sciences, Shenzhen, China

**Keywords:** folic acid, dosage, unmetabolized folic acid, H-type hypertension, safety

## Abstract

**Background:**

There is growing concern regarding elevated levels of circulating unmetabolized folic acid (UMFA) due to excessive intake of folic acid (FA). However, no randomized clinical trial has been conducted to examine the FA-UMFA dose-response relationship.

**Objective:**

This study aimed to investigate the FA-UMFA dose-response relationship in Chinese adults with hypertension and elevated homocysteine (H-type hypertension), a population with clear clinical indication for FA treatment.

**Methods:**

The data for this study were derived from a randomized, double-blind, multicenter clinical trial of 8 FA dosages on efficacy of homocysteine (Hcy) lowering. The parent trial had three 3 stages: screening period (2–10 days), run-in period (0–2 weeks, baseline visit), and double-blind treatment period (8 weeks) with follow-up visits at the end of the 2nd, 4th, 6th, and 8th weeks of treatment. Participants were randomly assigned to 8 treatment groups corresponding to FA dosages of 0, 0.4, 0.6, 0.8, 1.2, 1.6, 2.0 mg to 2.4 mg.

**Results:**

This study included 1,567 Chinese adults aged ≥45 years with H-type hypertension. There was a positive but non-linear association between FA supplementation and UMFA levels in the dosage range of 0 mg to 2.4 mg. In the regression analysis, the coefficients for the linear and quadratic terms of FA dosage were both statistically significant (*P* < 0.001). Notably, the slope for UMFA was greater for FA dosages >0.8 mg (ß = 11.21, 95% CI: 8.97, 13.45) compared to FA dosages ≤0.8 mg (ß = 2.94, 95% CI: 2.59, 3.29). Furthermore, FA dosages higher than 0.8 mg did not confer additional benefits in terms of increasing 5-methyl tetrahydrofolic acid (5-MTHF, active form of folate) or reducing homocysteine (Hcy).

**Conclusion:**

In Chinese adults with H-type hypertension, this study showed a positive, non-linear, dosage-response relationship between FA supplementation ranging from 0 to 2.4 mg and circulating UMFA levels. It revealed that 0.8 mg FA is an optimal dosage in terms of balancing efficacy (increasing 5-MTHF and lowering Hcy) while minimizing undesirable elevation of UMFA.

**Clinical trial registration:**

https://clinicaltrials.gov/ct2/show/NCT03472508?term=NCT03472508&draw=2&rank=1, identifier NCT03472508.

## Introduction

H-type hypertension, defined as essential hypertension with an increased plasma Hcy level (≥10 μmol/L), accounts for about 75% of hypertension among Chinese patients (1, 2). Many studies have indicated that FA supplementation can effectively lower blood pressure (BP), Hcy levels, and coagulation factors, and remarkably improves prothrombotic status in patients with H-type hypertension (3).

After absorption, however, FA needs to be reduced to tetrahydrofolate (THF) to activate its metabolism, subsequently further metabolized to 5-methyl tetrahydrofolic acid (5-MTHF), which is considered the most biologically active and functional form of FA. This activation process consists of two steps, both catalyzed by dihydrofolate reductase (DHFR). In the first step, FA is transformed into dihydrofolate (DHF); in the second step, DHF is transformed into THF (4). Notably, DHFR activity in humans is inefficient and easily saturated (5). Once the catalytic ability of DHFR has been saturated, UMFA accumulates in biological fluids including plasma. Early studies in adults showed that 0.2 mg of oral FA supplementation can lead to detectable concentrations of UMFA in the bloodstream; a daily dosage of 0.4 mg brought out a continuous occurrence of circulatory FA. However, newer, more sensitive methodologies have detected very low concentrations (∼0.8 nmol/L) of UMFA, even in people not taking FA supplementation (6). The presence of UMFA in the circulation is nearly ubiquitous, as it has been detected even in the cord blood of newly delivered infants (7).

Mandatory FA grain fortification in the US (starting in 1998) and in over 50 other countries has raised the circulating levels of UMFA in the general population (8, 9). This practice has also led to a growing concern regarding potential unintended adverse consequences due to high circulating UMFA from FA intake (10). NHANES (the National Health and Nutrition Examination Survey, a US representative sample), a study of elderly participants from 1999 to 2002, showed that UMFA was related to increased odds of anemia among participants who used alcohol (11), alterations in cytokine mRNA expression, a decreased number and weakened cytotoxicity of natural killer (NK) cells (12, 13), and cognitive impairment among seniors (14), as well as showed an association with insulin resistance and metabolic syndrome (15). However, uncertainty remains regarding the exact association between FA supplementation and circulating UMFA levels and what factors may modify the association.

The best study design to address this knowledge gap, is a randomized FA trial. Therefore, we conducted a study using data derived from a randomized, double-blind control, multicenter clinical trial (RCT) to delineate the dosage-response relationship between 8 different FA dosages (ranging from 0 to 2.4 mg daily by mouth) with circulating levels of UMFA. The original trial was conducted in H-type hypertension patients who all had elevated Hcy. As demonstrated in publications from our group (16) and others (17), FA supplementation is a well-established and effective treatment for this patient population. The goal of the current study was to identify the optimal FA dosage that would find the balance between maximizing efficacy in lowering Hcy and minimizing UMFA levels, while considering pertinent individual characteristics. This study represents a significant step toward a future vision of precision nutrition (18).

## Methods

### Study design

This study used data from a randomized, double-blind control, multicenter clinical study. The primary aim of the trial was to evaluate Hcy reduction efficacy by different dosages of FA among hypertension patients, stratified by MTHFR C677T genotypes (19).

The parent clinical trial consisted of 3 stages: (1) a screening period (2–10 days), (2) a run-in period (0–2 weeks), and (3) a double-blind treatment period (8 weeks). There were 6 study visits: the first at the beginning of the run-in period; the second at the beginning of the double-blind treatment period; and the third, fourth, fifth, and sixth at the end of the 2nd, 4th, 6th, and 8th weeks of treatment, respectively. Hypertension patients enrolled in the study who showed good tolerance for and compliance with angiotensin converting enzyme inhibitor (ACEI) drugs, and who had already been genotyped for the MTHFR C677T polymorphism during the run-in period, could directly enter the double-blind randomized treatment period. Drugs that could affect the efficacy evaluation were not permitted to be taken at any stage of the trial.

### Participants

Eligible participants were identified from patients presenting at hospitals located in the cities of Wuyuan, Anqing, and Lianyungang between March 2018 and June 2019. The inclusion criteria for the run-in period included patients aged 45 years or older who had been diagnosed with primary hypertension or were currently taking blood pressure medications; or for those who had not taken blood pressure medications within the past 2 weeks, had been newly diagnosed with hypertension, defined as diastolic blood pressure (DBP) ≥90 mmHg or systolic blood pressure (SBP) ≥140 mmHg; and Hcy ≥ 10 μmol/L. For these participants, two BP readings were taken, at least 1 day apart with the patient seated. Three measurements were taken at each visit, and the mean value of the three was used to determine hypertensive status at both visits. The 2nd BP was measured at visit 1. To qualify for the treatment period, patients had to have complete information on the detection of the MTHFR C677T gene polymorphism, and exhibit good tolerance to enalapril and good medication adherence (>80%).

Patients were excluded if they had secondary hypertension, cardiovascular diseases, digestive diseases (viral hepatitis, abnormal liver function, gastrointestinal dysfunction, etc.), urinary diseases, diabetes, corpulmonale, chronic obstructive pulmonary disease (COPD), stroke, malignancy, malnutrition, hematopoietic disorders and other serious diseases. Patients who were taking folate, B12 or B6, as well as those with frequent use of FA supplements or compounds containing FA within the previous 3 months were also excluded. Further exclusion criteria included anyone pregnant and/or lactating, and/or with an allergy or intolerance to enalapril and/or FA. Patients whose mental or nervous system dysfunction, inability to express desire, were excluded.

### Ethics approval

The clinical trial was carried out conforming to the Declaration of Helsinki, and the protocol was approved by the Ethics Committee of The Second Affiliated Hospital of Nanchang University Local Ethical Review process (February 4th, 2018) in China. Trial registration was completed before the beginning of recruitment (NCT03472508). All participants provided written, informed consent prior to any data collection. Supporting data will be provided by the corresponding author upon request, after consent from the Ethics Committee of The Second Affiliated Hospital of Nanchang University has been obtained.

### Allocation

**FIGURE 1:**
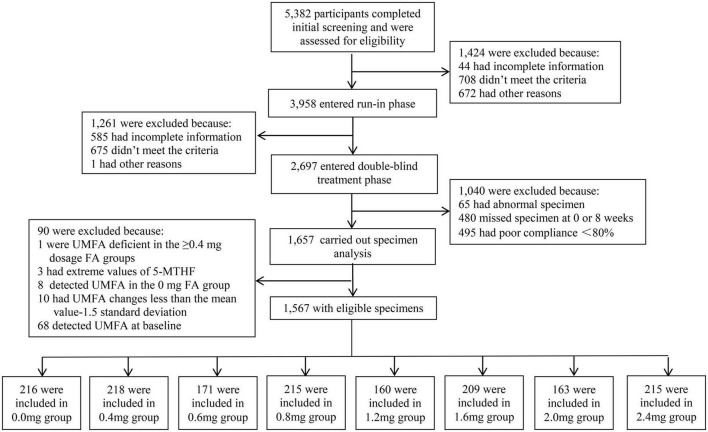
Flowchart of the study.

This study utilized data from a randomized, double blind, clinical trial in order to objectively evaluate the efficacy of different FA dosages on circulating UMFA levels, in addition to circulating folate and Hcy. A total of 5,382 patients were screened for the trial, and ultimately 2,697 patients entered into randomization and the double-blind treatment phase. All patients were first stratified by sex and the MTHFR C677T genotype (CC, CT, TT) for a total of six strata. Each of the six strata was then randomized into eight treatment groups according to a random list generated by SAS software, using quadratic block randomization as the randomization grouping method, consisting of either enalapril only (10 mg), or one of the other 7 treatment combinations with various dosages of FA (see Figure 1). All drugs were supplied by AUSA Pharmaceutical Limited; all test drugs were packed into safety capsules that appeared identical. Neither the clinicians/investigators nor the patients knew the contents of the capsules.

As shown in the study flow chart (Figure 1), there were 5,382 patients who completed the initial screening, and those who were ineligible for enrollment (*n* = 1,424) were disqualified. Although 2,697 patients entered into the randomization, only 1,657 patients had biospecimens for the lab analyses. The final sample for this study included 1,567 patients, of whom 218 patients received supplementation with 0.4 mg FA daily (Group 2), 171 with 0.6 mg FA daily (Group 3), 215 with 0.8 mg FA daily Group 4), 160 with 1.2 mg FA daily (Group 5), 209 with 1.6 mg FA daily (Group 6), 163 with 2 mg FA daily (Group 7), 215 with 2.4 mg FA daily (Group 8), and finally, 216 patients who received no FA supplementation who served as a control group (Group 1).

### Assessments

#### Sex, age, and BMI measures

Information on participant sex, age and body mass index (BMI) were collected and recorded at the baseline visit. Males and females were randomized to each group, in which all participants were aged over 45 years. BMI was calculated as the formula BMI = weight in kg/height in meters^2^.

#### Physical examination and lifestyle survey

Each participant completed a physical examination and questionnaire survey, covering lifestyle and disease history and medication use. All participants kept a diary throughout the study in which they reported their daily intake of capsules, any illnesses they experienced, and their use of medication. Habits of smoking and drinking were also recorded.

#### Biochemical measurements

To determine MTHFR C677T (rs1801133) polymorphisms, an ABI Prism 7900HT sequence detection system (Life Technologies) was used with the TaqMan assay. Detection of serum B12 levels collected at both the baseline visit during the run-in period and the double-blind treatment period was completed by chemiluminescent immunoassay in a commercial laboratory (New Industrial). Serum Hcy, creatinine, fasting lipids, estimated glomerular filtration rate (eGFR), and glucose levels at baseline were measured by automatic clinical analyzers (BeckmanCoulter). Quantification of UMFA and 5-MTHF in plasma was determined by stable-isotope dilution ultra-high performance liquid chromatography-tandem mass spectrometry. All the above measurements were conducted at the central laboratory of the Shenzhen Tailored Medical Laboratory, which obtained certificates of ISO9001:2015, ISO14001:2015, and ISO45001:2018.

### Statistical analysis

Continuous variables were presented as means ± SD or medians (IQR); and IQRs were expressed as 25th and 75th percentiles. Categorical variables were presented as numbers (%). The absolute changes in UMFA levels from values at baseline to values at the 8th week of the intervention were calculated. The differences in baseline characteristics between those receiving different dosages of FA in the intervention group were compared by ANOVA tests or Chi square tests. After adjusting for covariables, changes in UMFA for each of the different dosages were determined by graphical smoothing curves using a generalized linear model. The smoothing and regression models were adjusted for center, sex, age, BMI, smoking and drinking status, C677T, baseline eGFR, baseline fasting glucose (GLU), baseline high density lipoprotein cholesterol (HDL-C), baseline total cholesterol (TC), baseline triglycerides (TG), SBP, and DBP. The additive effects of the linear and quadratic terms of the model were tested using chi-square tests (degrees of freedom: 2). The interaction between subgroups of each parameter and FA supplement dosage was assessed by the Wald test, which was used for measuring interactions on a multiplicative scale. Threshold analysis in the correlation of serum folate with UMFA levels was done with a 2-piecewise Cox regression model by a smoothing function, with cutoffs at ≤0.8 and >0.8 mg/day. A likelihood-ratio test and bootstrap resampling methods were employed to confirm the threshold level (i.e., inflection point). All analysis of data were conducted using the statistical package R^1^ and Stata software, version 14.0 (Stata Corp). A two-tailed *P* < 0.05 was supposed to have statistical significance.

## Results

Many of the baseline characteristics between the eight dosage treatment groups (Table 1) were quite similar, including the MTHFR C677T genotype, baseline folate levels, and Hcy levels. Compared with the control group, the exit folate and exit UMFA levels in the FA groups were significantly higher, and these increased with increasing FA dosage. In comparing eligible patients with ineligible patients, high-density lipoprotein cholesterol (HDL-C) and exit Hcy (*p* < 0.001) differed significantly (Supplementary Table 1).

**TABLE 1:** Baseline characteristics of study participants^a^.

FA dosage	0 mg	0.4 mg	0.6 mg	0.8 mg	1.2 mg	1.6 mg	2 mg	2.4 mg	*P*-value^b^
Participants (*n*)	216	218	171	215	160	209	163	215	
**Center**									0.194
Anqing	42 (19.4)	39 (17.9)	32 (18.7)	42 (19.5)	30 (18.8)	42 (20.1)	28 (17.2)	48 (22.3)	
Lianyungang	73 (33.8)	79 (36.2)	49 (28.7)	77 (35.8)	43 (26.9)	76 (36.4)	40 (24.5)	73 (34.0)	
Wuyuan	101 (46.8)	100 (45.9)	90 (52.6)	96 (44.7)	87 (54.4)	91 (43.5)	95 (58.3)	94 (43.7)	
Male	97 (44.9)	103 (47.2)	86 (50.3%)	102 (47.4)	75 (46.9)	96 (45.9)	87 (53.4)	101 (47.0)	0.823
Age, y	65.5 ± 8.1	63.9 ± 7.7	65.1 ± 7.6	65.0 ± 7.9	64.5 ± 8.1	64.6 ± 7.4	64.0 ± 9.0	64.8 ± 9.0	0.527
BMI, kg/m^2^	24.9 ± 6.9	24.9 ± 3.5	25.3 ± 11.3	24.9 ± 3.5	24.2 ± 3.5	24.4 ± 3.4	24.7 ± 3.2	24.6 ± 3.4	0.655
**Smoking**									0.496
Never	140 (64.8)	144 (66.1)	115 (67.3)	131 (60.9)	100 (62.5)	136 (65.1)	95 (58.3)	144 (67.0)	
Former	31 (14.4)	23 (10.6)	25 (14.6)	26 (12.1)	22 (13.8)	28 (13.4)	32 (19.6)	24 (11.2)	
Current	45 (20.8)	51 (23.4)	31 (18.1)	58 (27.0)	38 (23.8)	45 (21.5)	36 (22.1)	47 (21.9)	
Drinking									0.875
Never	135 (62.8)	138 (63.3)	111 (64.9)	140 (65.1)	109 (68.1)	136 (65.1)	112 (68.7)	145 (67.4)	
Former	30 (14.0)	20 (9.2)	23 (13.5)	23 (10.7)	15 (9.4)	28 (13.4)	17 (10.4)	24 (11.2)	
Current	50 (23.3)	60 (27.5)	37 (21.6)	52 (24.2)	36 (22.5)	45 (21.5)	34 (20.9)	46 (21.4)	
**C677T**									0.861
CC	64 (29.6)	64 (29.4)	55 (32.2)	67 (31.2)	53 (33.1)	57 (27.3)	48 (29.4)	75 (34.9)	
CT	105 (48.6)	105 (48.2)	81 (47.4)	98 (45.6)	70 (43.8)	96 (45.9)	80 (49.1)	85 (39.5)	
TT	47 (21.8)	49 (22.5)	35 (20.5)	50 (23.3)	37 (23.1)	56 (26.8)	35 (21.5)	55 (25.6)	
eGFR, mL/min/1.73 m^2^	96.6 (88.0, 104.3)	98.0 (88.8, 104.5)	95.2 (86.8, 104.2)	96.4 (87.8, 104.4)	98.2 (86.4, 107.6)	96.6 (89.1, 104.4)	94.5 (83.9, 104.3)	95.7 (87.9, 105.7)	0.803
GLU, mmol/L	5.7 (5.3, 6.4)	5.8 (5.3, 6.3)	5.7 (5.4, 6.3)	5.9 (5.4, 6.4)	5.8 (5.4, 6.2)	5.8 (5.3, 6.4)	5.9 (5.3, 6.5)	5.7 (5.4, 6.2)	0.670
Hcy, μmol/L	14.4 (11.8, 17.8)	14.3 (12.3, 17.3)	14.6 (11.9, 17.6)	14.1 (12.1, 17.9)	13.3 (11.4, 17.3)	14.5 (11.9, 17.5)	14.3 (12.0, 17.5)	14.5 (12.2, 17.5)	0.897
HDL-C, mmol/L	1.8 (1.4, 2.1)	1.6 (1.4, 1.9)	1.7 (1.4, 2.1)	1.7 (1.4, 2.1)	1.7 (1.4, 1.9)	1.7 (1.4, 2.1)	1.6 (1.4, 1.9)	1.7 (1.4, 2.0)	0.243
TC, mmol/L	5.5 (4.7, 6.1)	5.2 (4.7, 5.9)	5.3 (4.8, 6.1)	5.2 (4.6, 5.9)	5.4 (4.6, 5.9)	5.3 (4.6, 6.0)	5.3 (4.7, 5.9)	5.3 (4.8, 5.9)	0.810
TG, mmol/L	1.4 (1.0, 2.0)	1.4 (1.0, 2.2)	1.6 (1.1, 2.1)	1.4 (1.0, 2.0)	1.4 (1.0, 2.2)	1.4 (1.0, 2.0)	1.4 (1.0, 2.1)	1.4 (1.0, 2.0)	0.703
Folate, ng/mL	12.3 (7.8, 16.9)	10.6 (7.2, 16.6)	11.2 (8.0, 16.3)	10.4 (7.1, 14.5)	12.4 (8.4, 17.8)	10.9 (7.7, 16.4)	11.3 (8.4, 17.3)	11.1 (7.3, 16.5)	0.055
Exit folate, ng/mL	10.1 (7.2, 14.8)	26.9 (17.7, 40.3)	40.7 (23.4, 56.7)	50.0 (25.3, 81.5)	86.1 (32.2, 148.2)	97.5 (23.9, 194.9)	168.8 (45.4, 313.7)	153.6 (40.3, 312.3)	<0.001
Change in folate, ng/mL	−0.8 (−5.8, 1.5)	14.3 (7.0, 26.0)	26.3 (12.1, 44.5)	37.5 (15.0, 69.6)	67.4 (18.5, 132.2)	84.5 (15.0, 177.5)	158.1 (36.0, 299.8)	139.9 (25.8, 303.4)	<0.001
Exit UMFA, ng/mL	0.2 (0.2, 0.2)	1.1 (0.2, 2.9)	3.5 (0.7, 7.1)	6.2 (0.2, 15.1)	20.4 (0.9, 30.8)	27.3 (0.5, 51.3)	41.6 (3.9, 70.0)	54.0 (5.3, 84.9)	<0.001
Change in UMFA, ng/mL	0.0 (0.0, 0.0)	0.9 (0.0, 2.7)	3.2 (0.4, 6.9)	6.0 (0.0, 14.9)	20.2 (0.6, 30.5)	27.0 (0.3, 51.1)	41.3 (3.7, 69.7)	53.8 (5.0, 84.7)	<0.001

^a^Values are the means ± SD, median (interquartile range), and number (percentage) for variables with normal distribution, variables with skewed distribution, and categorical variables, respectively. ^b^Among-group differences were compared using the ANOVA test and the χ 2 test for continuous variables and categorical variables, respectively. BMI, body mass index; C677T, MTHFR C677T genotype; eGFR, estimated glomerular filtration rate; FA, folic acid; GLU, fasting glucose; Hcy, homocysteine; HDL-C, high-density lipoprotein cholesterol; TC, total cholesterol; TG, triglycerides; UMFA, unmetabolized folic acid.

**FIGURE 2:**
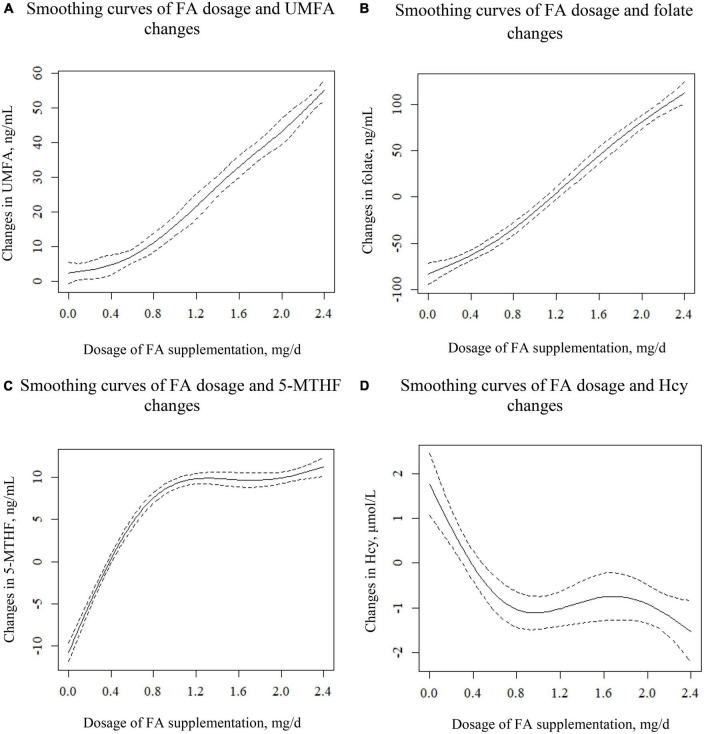
Smoothing curves illustrating the association between different dosages of FA and the changes in UMFA **(A)**, folate **(B)**, 5-MTHF **(C)**, and Hcy **(D)**. Adjusted for center, sex, age, BMI, smoking and drinking status, C677T, baseline eGFR, baseline GLU, baseline HDL-C, baseline TC, baseline TG, SBP, and DBP.

Figure 2 shows the smoothing curves illustrating the association between different dosages of FA and changes in UMFA (a); folate (b); 5-MTHF (c); and Hcy (d), indicating 0.8 mg/day as the critical FA dosage for stimulating UMFA change. As shown in Figure 2A, as FA dosage increased from 0 to 2.4 mg/day, change in UMFA increased continuously: for FA dosages higher than 0.8 mg/day, UMFA jumped dramatically, while for FA dosages lower than 0.8 mg/day, change in UMFA tended to increase at a much slower rate.

As shown in Figure 2B, changes in folate displayed a positively curved relationship with different dosages of FA. For FA dosages higher than 0.8 mg/day, the slope of the curve was slightly greater than for FA dosages lower than 0.8 mg/day.

As shown in Figure 2C, as the FA dosage increased from 0 to 0.8 mg, changes in 5-MTHF levels rose sharply to 10 ng/mL, after which it remained nearly unchanged. For FA dosages higher than 0.8 mg, the 5-MTHF level appeared to level off at 10 ng/mL.

As shown in Figure 2D, after a sharp initial plunge, the curve flattened out at FA dosage 0.8 mg, and then fell again at FA dosage 1.6 mg. Specifically, as the FA dosage increased from 0 to 0.8 mg, change in Hcy decreased from 2 to −1 μmol/L; and with an FA dosage between 0.8 and 1.6 mg, change in Hcy remained constant at −1 μmol/L, while an FA dosage above 1.6 mg led to a Hcy reduction of −1 to −2 μmol/L.

Combined with Figure 2A, the threshold effect analysis on the effect of FA dosage on UMFA is shown in Table 2. The results of the analysis indicated a positive correlation between the two. All patients were divided into two groups according to FA supplementation with 0.8 mg/day as a new grouping point: low-dosage (≤0.8 mg) and high-dosage FA group (>0.8 mg). For each 0.2, or 0.4 mg/day FA dosage increase, the slope for UMFA was greater for the high-dosage FA group (ß = 11.21, 95% CI (8.97, 13.45) compared to the low-dosage FA group (ß = 2.94, 95% CI: 2.59, 3.29) (*P* for interaction, <0.001). The coefficients for the linear and quadratic terms for FA dosages were both statistically significant (*P* < 0.001) in the total sample regression analysis (Supplementary Table 2).

**TABLE 2:** Threshold effect analysis: the dose-response relationship of folic acid supplementation with circulating unmetabolized folic acid (UMFA), stratified by FA dosage subgroups (≤ 0.8 vs. >0.8 mg/day)^a^.

FA dosage* mg/day	Crude model		Adjusted model		*P* for interaction
	**β (95% CI)**	***P-*value**	**β (95% CI)**	***P-*value**	
≤0.8	2.93 (2.58, 3.29)	<0.001	2.94 (2.59, 3.29)	<0.001	<0.001
>0.8	11.09 (8.77, 13.41)	<0.001	11.21 (8.97, 13.45)	<0.001	

^a^Adjusted for center, sex, age, BMI, smoking and drinking status, C677T, baseline eGFR, baseline GLU, baseline HDL-C, baseline TC, baseline TG, SBP, and DBP.

*FA dosage: ≤ 0.8 mg/day (0, 0.4, 0.6, 0.8); FA dosage: >0.8 mg/day (1.2, 1.6, 2.0, 2.4 mg/day).

Table 3 shows the results of a stratified analysis of the effect of FA dosage on UMFA. For those in the low-dosage FA group (≤0.8 mg/day), those with a lower BMI showed a stronger association between FA dosage and UMFA (< 24.5 vs. ≥ 24.5 kg/m^2^; *P* for interaction, <0.001). In addition, hospital center (*p* for interaction, <0.001) and smoking status (*p* for interaction, 0.019) positively modified the association between FA dosage and UMFA levels in the low-dosage group, with a stronger correlation found among current smokers and patients from Anqing or Wuyuan. In the high-dosage FA group (1.2–2.4 mg/day) none of the other variables, including age, sex, hospital center, BMI, SBP, DBP, TC, triglycerides, HDL-C, GLU, eGFR, MTHFR C677T, rs70991108 (a polymorphism consisting of a 19-bp deletion in the first intron of the DHFR gene), smoking status, or drinking status, significantly modified the relationship between FA dosage and UMFA levels.

**TABLE 3:** Stratified analyses by participant characteristics on the effect of folic acid dosage on unmetabolized folic acid (UMFA)^a^.

Subgroup	FA dosage: ≤ 0.8 mg/day (0, 0.4, 0.6, 0.8 mg)				FA dosage: >0.8 mg/day (1.2, 1.6, 2.0, 2.4 mg)			
	***N***	**UMFA, ng/ml Mean ± SD**	**β (95% CI)**	***P* for interaction**	***N***	**UMFA, ng/ml Mean ± SD**	**β (95% CI)**	***P* for interaction**
Center				<0.001				0.144
Anqing	155	4.5 ± 7.7	3.31 (2.39, 4.23)		148	45.2 ± 36.5	15.07 (10.25, 19.89)	
Lianyungang	278	2.6 ± 5.9	1.67 (1.08, 2.25)		232	23.0 ± 31.4	8.78 (5.17, 12.4)	
Wuyuan	387	4.8 ± 6.9	3.69 (3.2, 4.17)		367	43.0 ± 40.4	11.24 (7.66, 14.83)	
Sex				0.187				0.694
Male	388	4.0 ± 6.3	3.19 (2.74, 3.65)		359	36.8 ± 32.9	11.78 (9.04, 14.51)	
Female	432	4.2 ± 7.1	2.74 (2.21, 3.27)		388	36.8 ± 42.6	10.56 (7.07, 14.06)	
Age, year				0.089				0.264
<65.4 (median)	409	3.6 ± 6.6	2.64 (2.12, 3.16)		383	33.3 ± 33.8	9.91 (7, 12.82)	
≥65.4	411	4.4 ± 6.9	3.26 (2.78, 3.75)		364	41.0 ± 41.9	12.31 (8.84, 15.77)	
BMI, kg/m^2^				<0.001				0.979
<24.5 (median)	406	4.8 ± 7.6	3.59 (3.04, 4.13)		390	41.9 ± 37.1	11.34 (8.26, 14.43)	
≥24.5	414	3.5 ± 5.9	2.33 (1.88, 2.77)		357	32.6 ± 38.9	10.65 (7.31, 13.99)	
Smoking				0.019				0.621
Never	530	4.0 ± 6.7	2.66 (2.21, 3.12)		475	39.1 ± 41.4	11.52 (8.48, 14.57)	
Former	105	3.2 ± 4.9	2.5 (1.80, 3.19)		106	35.8 ± 35.7	11.53 (5.57, 17.5)	
Current	185	5.0 ± 7.7	3.74 (2.94, 4.53)		166	33.8 ± 28.9	9.75 (6.14, 13.35)	
Drinking				0.562				0.619
Never	524	4.3 ± 7.1	2.8 (2.34, 3.27)		502	37.4 ± 40.2	10.55 (7.65, 13.45)	
Former	96	3.7 ± 5.7	3.16 (2.29, 4.03)		84	37.6 ± 35.9	13.09 (6.24, 19.94)	
Current	199	3.8 ± 6.4	3.12 (2.46, 3.78)		161	36.4 ± 32.9	11.19 (7.01, 15.37)	
MTHFR C677T				0.175				0.352
CC	250	4.1 ± 7.0	3.23 (2.56, 3.9)		233	38.6 ± 42.3	9.5 (5.02, 13.99)	
CT	389	4.2 ± 7.0	3.05 (2.52, 3.58)		331	36.3 ± 36.0	13.03 (9.81, 16.26)	
TT	181	4.0 ± 5.9	2.26 (1.6, 2.93)		183	37.9 ± 36.7	9.61 (5.12, 14.1)	
rs70991108				0.346				0.922
-/-	250	3.7 ± 6.2	2.61 (2.09, 3.12)		178	43.5 ± 41.0	13.07 (7.16, 18.98)	
±	351	4.2±7.1	3.11 (2.53, 3.69)		281	39.1±42.7	11 (6.71, 15.3)	
+ /+	70	4.0 ± 7.8	3.59 (1.99, 5.19)		45	31.5 ± 36.2	9.62 (−5.46, 24.69)	
eGFR, mL/min per 1.73 m^2^				0.085				0.875
<96.5 (median)	411	4.7 ± 7.4	3.17 (2.63, 3.72)		379	41.1 ± 41.2	10.96 (7.41, 14.51)	
≥96.5	407	3.6 ± 6.1	2.59 (2.14, 3.03)		368	33.7 ± 34.5	11.39 (8.54, 14.23)	
GLU, mmol/L				0.058				0.647
<5.8 (median)	416	4.3 ± 6.9	3.29 (2.8, 3.78)		372	37.4 ± 35.7	11.94 (9, 14.89)	
≥5.8	402	4.0 ± 6.7	2.62 (2.11, 3.13)		375	37.4 ± 40.6	10.74 (7.25, 14.23)	
HDL-C, mmol/L				0.368				0.425
<1.7 (median)	421	3.8 ± 6.6	2.77 (2.26, 3.28)		398	34.4 ± 34.7	10.38 (7.58, 13.18)	
≥1.7	397	4.5 ± 6.9	3.08 (2.58, 3.58)		349	40.4 ± 41.6	11.96 (8.31, 15.62)	
TC, mmol/L				0.796				0.982
<5.3 (median)	406	4.0 ± 6.5	3.03 (2.56, 3.5)		362	36.9 ± 40.5	11.28 (7.79, 14.76)	
≥5.3	412	4.3 ± 7.0	2.93 (2.4, 3.46)		385	37.9 ± 36.0	11.18 (8.24, 14.12)	
TG, mmol/L				0.845				0.665
<1.4 (median)	397	4.1 ± 6.7	2.94 (2.45, 3.42)		367	39.3 ± 41.0	11.49 (8.07, 14.91)	
≥1.4	421	4.2 ± 6.9	2.9 (2.37, 3.42)		380	35.6 ± 35.2	10.35 (7.42, 13.28)	
DBP, mmHg				0.154				0.884
<90.0 (median)	409	4.4 ± 6.8	3.21 (2.72, 3.71)		367	39.3 ± 41.4	11.01 (7.54, 14.47)	
≥90.0	411	3.8 ± 6.7	2.69 (2.18, 3.2)		380	35.6 ± 34.7	11.39 (8.54, 14.24)	
SBP, mmHg				0.101				0.401
<151.0 (median)	407	3.8 ± 6.8	2.64 (2.1, 3.19)		356	39.6 ± 41.4	10.19 (6.51, 13.87)	
≥151.0	413	4.5 ± 6.7	3.24 (2.78, 3.69)		391	35.5 ± 35.0	12.13 (9.36, 14.91)	

^a^Adjusted for center, sex, age, BMI, smoking and drinking status, C677T, baseline eGFR, baseline GLU, baseline HD-CL, baseline TC, baseline TG, SBP, and DBP.

## Discussion

To our knowledge, this is the largest study utilizing data from a RCT to assess the dosage-response relationship between FA supplementation (eight different FA dosages) and circulating levels of UMFA, and is the first such study in a Chinese population. The results of this research provide critical insights into the optimal dosage of FA supplementation while balancing efficacy and adverse effects. These findings offer practical implications for public health professionals and clinicians.

Our research results show that high intake of FA can lead to the occurrence of UMFA in plasma, which is consistent with that of several previous studies. A study in an elderly Irish cohort of 137 participants with a mean age of 67.4 years demonstrated that an oral FA dosage above 200 μg (threshold dosage) resulted in UMFA in plasma (20). Stamm et al. reported on a study of 117 women who received a daily multivitamin containing 1000 μg FA throughout pregnancy and lactation until 8 weeks postpartum, at which point, UMFA was detected in nearly all non-fasted blood samples (21). Sweeney et al. tested the postprandial serum FA response to multiple dosages of FA in fortified bread and found the appearance of UMFA in the serum of all participants at all test dosages, showing apparent accumulation effects (22). However, these results only proved that higher intake of FA leads to higher occurrence of UMFA in the serum, without identifying which dosage of FA is the optimum, and offered no practical guiding significance for clinical application.

An understanding of the absorption and metabolism of FA in humans can reveal the reasons for the occurrence of UMFA in the serum. Previous studies have shown that FA is mainly absorbed in the proximal jejunum in a prototype form after oral administration, and oral doses of FA in excess of about 260–280 μg (589–634 nmol) leads to the direct appearance of UMFA in the systemic circulation (6). Absorbed folate, which may undergo biotransformation in the absorptive mucosa, is subsequently transferred via the mesenteric veins to the hepatic portal vein and carried to the liver where an extensive amount (liver “first-pass”) is removed (23). While the liver has a high affinity for the removal of FA, it has a low affinity for the removal of 5-MTHF (24). This may have important implications for FA use as a supplement or fortification since the human liver has a low capacity for reduction and may eventually give rise to saturation, resulting in significant (and potentially deleterious) UMFA entering systemic circulation (25). Our study investigated the saturation point of oral FA transformation through two indicators, namely, folate and 5-MTHF, as well as the saturation point of FA on lowering Hcy levels. Our study also identified that when the FA dosage is equal to 0.8 mg/day, the biotransformation capacity of FA into 5-MTHF in humans is saturated, and the effect of FA on reducing Hcy is also basically saturated.

This study also revealed individual characteristics that may modify the FA supplementation and UMFA association. There was a significant interaction between FA supplementation dosage and age on UMFA levels (Supplementary Table 3). Compared with participants aged ≥65.4 years, significantly lower UMFA levels (mean: 17.9 vs. 21.6, *p* < 0.001) were found among those aged <65.4 years. It is likely that aging may slow down the absorption and metabolism of FA: as we age the older the age, the weaker the FA absorption declines, and metabolism weakens and the higher the UMFA levels increase. M Wolters et al. considered that atrophic gastritis could result in declining gastric acid and pepsinogen secretion, and hence decreasing the intestinal digestion and absorption of both B vitamins (i.e., vitamin B12 and FA), wherein atrophic gastritis occurred with a frequency of approximately 20–50% in the elderly subjects (26). There was also a significant interaction effect between FA supplementation and BMI on UMFA levels. Compared with participants with BMI ≥ 24.4, significantly higher UMFA levels were found among those with BMI < 24.4 (Mean: 22.8 vs. 16.7, *p* < 0.001). This indicates that people of different BMI have different FA requirements, which is consistent with the results reported in the previous literature (27). One explanation for this observation is that, as body size increases, the distribution of folate changes, resulting in changes of freely available plasma/serum folate and folate in the cells (28, 29).

Overall, our study provides a significant contribution to the literature on FA supplementation and UMFA levels in hypertensive adults. The research findings have several practical implications for clinicians, public health professionals, and policymakers for developing more effective interventions and strategies to reduce the risk of UMFA accumulation and its potential adverse health outcomes.

The research highlights the importance of regularly monitoring FA supplementation in patients with hypertension. However, attention should be paid to the limitations of the present study. We did not compare the correlation between FA intake and circulating UMFA among participants from different racial groups or ethnicities or for individuals under 45 years of age. It is important for future research to explore the optimal dosage of FA supplementation in other populations with different characteristics to investigate the effects of long-term, high-dosage FA intake on UMFA levels and related health outcomes.

## Conclusion

This study, utilizing data from a large, randomized nutrition trial, showed a positive, non-linear, dosage-response relationship between FA supplementation ranging from 0 mg to 2.4 mg and circulating UMFA levels in Chinese adults with H-type hypertension. Our findings indicate that, on average, supplementation with 0.8 mg/day FA appears to be an optimal dosage in balancing efficacy vs. UMFA levels.

## Data availability statement

The raw data supporting the conclusions of this article will be made available by the authors, without undue reservation.

## Ethics statement

The studies involving humans were approved by the Ethics Committee of The Second Affiliated Hospital of Nanchang University. The studies were conducted in accordance with the local legislation and institutional requirements. Written informed consent for participation in this study was provided by the participants’ legal guardians/next of kin.

## Author contributions

PC, JJ, SL, XQ, and XX designed the research. PC, YS, NZ, YW, ZZ, QH, BW, LL, XH, XC, GT, YD, and HZ conducted the research. PC, XQ, SS, and ZZ collected and analyzed the data. PC, LT, ZZ, and QH drafted the manuscript. All authors read and approved the final manuscript.

## Footnotes

1
http://www.r-project.org
