# Supplementary material for: Association of folic acid dosage with circulating unmetabolized folic acid in Chinese adults with H-type hypertension: a multicenter, double-blind, randomized controlled trial
Source: Front Nutr. 2023 Sep 14;10:1191610. doi: 10.3389/fnut.2023.1191610 (PMC10538967; doi:10.3389/fnut.2023.1191610)
Supplement: Supplementary file 1 [file Data_Sheet_1.docx]

**Supplementary Table 1**. Comparison of baseline characteristics of the included and excluded populations^a^.

|  | Excluded Population | Included Population | *P^b^* |
| --- | --- | --- | --- |
| Individual (n) | 90 | 1567 |  |
| Male | 52 (57.8) | 747 (47.7) | 0.062 |
| Age, y | 67.3 ± 7.8 | 64.7 ± 8.1 | 0.003 |
| BMI, kg/m^2^ | 23.9 ± 3.1 | 24.8 ± 5.4 | 0.139 |
| Smoking |  |  | 0.002 |
| Never | 48 (53.3) | 1005 (64.1) |  |
| Former | 24 (26.7) | 211 (13.5) |  |
| Current | 18 (20.0) | 351 (22.4) |  |
| Drinking |  |  | 0.036 |
| Never | 47 (52.2) | 1026 (65.5) |  |
| Former | 15 (16.7) | 180 (11.5) |  |
| Current | 28 (31.1) | 360 (23.0) |  |
| C677T |  |  | 0.033 |
| CC | 30 (33.3) | 483 (30.8) |  |
| CT | 30 (33.3) | 720 (45.9) |  |
| TT | 30 (33.3) | 364 (23.2) |  |
| GLU, mmol/L | 4.8 (4.2-5.6) | 5.3 (4.7-5.9) | 0.003 |
| Hcy, μmol/L | 14.6 (12.2-17.7) | 13.5 (11.1-16.3) | 0.029 |
| Exit Hcy, μmol/L | 13.5 (12.1-15.9) | 11.6 (9.8-14.0) | <0.001 |
| HDL-C, mmol/L | 1.6 (1.4-1.9) | 1.4 (1.1-1.7) | <0.001 |
| folate, ng/mL | 9.8 (7.0-16.6) | 11.3 (7.5-16.5) | 0.361 |
| Exit folate, ng/mL | 45.6 (19.1-121.8) | 41.4 (18.4-129.6) | 0.910 |
| TC, mmol/L | 5.1 (4.5-5.8) | 5.1 (4.5-5.7) | 0.657 |
| TG, mmol/L | 1.3 (0.9-1.7) | 1.4 (1.0-2.0) | 0.126 |

^a^ Values are the means ± SD, median (interquartile range), and number (percentage) for variables with normal distribution, variables with skewed distribution, and categorical variables, respectively. Abbreviations: BMI, body mass index; C677T, MTHFR C677T genotype; GLU, fasting glucose; Hcy, homocysteine; HDL-C, high-density lipoprotein cholesterol; TC, total cholesterol; TG, triglycerides.

*^b^* Among-group differences were compared using the ANOVA test and the χ 2 test for continuous variables and categorical variables, respectively.

**Supplementary Table 2**. Association between different dosages of FA and the changes of UMFA^a^.

|  | N | UMFA,ng/ml | Crude Model | Adjusted Model |
| --- | --- | --- | --- | --- |
|  |  | Mean (SD) | β (SE), *P* | β (SE), *P* |
| **All participants** | 1567 | 20.0 (31.6) |  |  |
| Dosage |  |  | 10.64 (3.01), <0.001 | 10.27 (2.96), <0.001 |
| Dosage^2^ |  |  | 5.30 (1.19), <0.001 | 5.37(1.17), <0.001 |
| Chi-square value |  |  | 861.734 | 887.411 |
| *P* for Joint effect (df=2) |  |  | <0.001 | <0.001 |

^a^ Adjusted for center, sex, age, BMI, smoking and drinking status, C677T, baseline eGFR, baseline GLU, baseline HDL-C, baseline TC, baseline TG, SBP, and DBP.

**Supplementary Table 3**. Stratified analysis between different dosages of FA and the changes of UMFA^a^.

| Subgroups | n | UMFA,ng/ml | Adjusted Model | *P* for interaction | 　$x^{2}$ | df | Joint effect |
| --- | --- | --- | --- | --- | --- | --- | --- |
|  |  | Mean (SD) | β（SE) , *P* |  |  |  |  |
| **Center** |  |  |  |  |  |  |  |
| Anqing |  |  |  |  |  |  |  |
| Dosage | 303 | 24.2 (33.0) | 5.97 (6.26) 0.341 | <0.001 | 306.795 | 2 | <0.001 |
| Dosage^2^ |  |  | 9.24 (2.45) <0.001 |  |  |  |  |
| Lianyungang |  |  |  |  |  |  |  |
| Dosage | 510 | 11.9 (23.9) | 1.39 (4.28) 0.745 | ref | 170.894 | 2 | <0.001 |
| Dosage^2^ |  |  | 5.52 (1.70) 0.001 |  |  |  |  |
| Wuyuan |  |  |  |  |  |  |  |
| Dosage^b^ | 754 | 23.3 (34.5) | 19.34 (4.76) <0.001 | <0.001 | 444.284 | 2 | <0.001 |
| Dosage^2^ |  |  | 3.25 (1.89) 0.086 |  |  |  |  |
| **Sex** |  |  |  |  |  |  |  |
| Male |  |  |  |  |  |  |  |
| Dosage | 747 | 19.5 (28.4) | 9.16 (3.70) 0.014 | 0.677 | 572.367 | 2 | <0.001 |
| Dosage^2^ |  |  | 5.76 (1.46) <0.001 |  |  |  |  |
| Female |  |  |  |  |  |  |  |
| Dosage | 820 | 20.0 (34.2) | 11.07 (4.53) 0.015 | ref | 360.178 | 2 | <0.001 |
| Dosage^2^ |  |  | 5.07 (1.80) 0.005 |  |  |  |  |
| **Age, y** |  |  |  |  |  |  |  |
| B1(<65.4) | 785 |  |  |  |  |  |  |
| Dosage |  | 17.9 (28.2) | 9.48 (3.89) 0.015 | <0.001 | 398.017 | 2 | <0.001 |
| Dosage^2^ |  |  | 4.61 (1.54) 0.003 |  |  |  |  |
| B2(≥65.4) | 782 |  |  |  |  |  |  |
| Dosage |  | 21.6(34.5) | 11.76(4.48) 0.009 | ref | 471.482 | 2 | <0.001 |
| Dosage^2^ |  |  | 5.82(1.76) 0.001 |  |  |  |  |
| **BMI, kg/m^2^** |  |  |  |  |  |  |  |
| B1(<24.4) |  |  |  |  |  |  |  |
| Dosage | 784 | 22.8 (32.5) | 15.67(4.10) <0.001 | <0.001 | 544.227 | 2 | <0.001 |
| Dosage^2^ |  |  | 4.27 (1.64) 0.009 |  |  |  |  |
| B2(≥24.4） |  |  |  |  |  |  |  |
| Dosage | 783 | 16.7(30.3) | 4.72 (4.27) 0.270 | ref | 337.874 | 2 | <0.001 |
| Dosage^2^ |  |  | 6.47 (1.68) <0.001 |  |  |  |  |
| **Smoking** |  |  |  |  |  |  |  |
| Never |  |  |  |  |  |  |  |
| Dosage | 1005 | 20.3 (33.8) | 9.58 (3.99) 0.016 | ref | 531.922 | 2 | <0.001 |
| Dosage2 |  |  | 6.07 (1.58) <0.001 |  |  |  |  |
| Former |  |  |  |  |  |  |  |
| Dosage | 211 | 19.3 (30.3) | 5.00 (7.76) 0.520 | 0.465 | 135.617 | 2 | <0.001 |
| Dosage^2^ |  |  | 7.80 (3.15) 0.014 |  |  |  |  |
| Current |  |  |  |  |  |  |  |
| Dosage | 351 | 18.4 (25.1) | 15.30 (4.91) 0.002 | 0.018 | 240.305 | 2 | <0.001 |
| Dosage^2^ |  |  | 2.16 (1.93) 0.264 |  |  |  |  |
| **Drinking** |  |  |  |  |  |  |  |
| Never |  |  |  |  |  |  |  |
| Dosage | 1026 | 20.4 (33.1) | 11.24 (3.95) 0.005 | ref | 493.158 | 2 | <0.001 |
| Dosage^2^ |  |  | 4.90 (1.56) 0.002 |  |  |  |  |
| Former |  |  |  |  |  |  |  |
| Dosage | 180 | 19.4 (30.2) | 4.82 (7.81) 0.538 | 0.768 | 124.678 | 2 | <0.001 |
| Dosage^2^ |  |  | 7.92 (3.12) 0.012 |  |  |  |  |
| Current |  |  |  |  |  |  |  |
| Dosage | 360 | 18.1 (27.7) | 10.32 (5.15) 0.046 | 0.997 | 279.37 | 2 | <0.001 |
| Dosage^2^ |  |  | 5.28 (2.05) 0.010 |  |  |  |  |
| **C677T** |  |  |  |  |  |  |  |
| CC |  |  |  |  |  |  |  |
| Dosage | 483 | 20.5 (34.4) | 16.68 (6.17) 0.007 | 0.833 | 210.228 | 2 | <0.001 |
| Dosage^2^ |  |  | 2.85 (2.40) 0.236 |  |  |  |  |
| CT |  |  |  |  |  |  |  |
| Dosage | 720 | 18.7 (29.6) | 4.72 (3.97) 0.234 | 0.280 | 492.489 | 2 | <0.001 |
| Dosage^2^ |  |  | 7.84 (1.60) <0.001 |  |  |  |  |
| TT |  |  |  |  |  |  |  |
| Dosage | 364 | 20.8 (31.3) | 14.04 (6.14) 0.023 | ref | 183.950 | 2 | <0.001 |
| Dosage^2^ |  |  | 3.51 (2.41) 0.147 |  |  |  |  |
| **eGFR, ml/min/1.73m^2^** |  |  |  |  |  |  |  |
| B1 (<96.4) |  |  |  |  |  |  |  |
| Dosage | 782 | 22.0 (34.4) | 14.62 (4.70) 0.002 | 0.015 | 406.854 | 2 | <0.001 |
| Dosage^2^ |  |  | 4.24 (1.85) 0.022 |  |  |  |  |
| B2 (≥96.4） |  |  |  |  |  |  |  |
| Dosage | 783 | 17.6 (28.4) | 6.64 (3.66) 0.070 | ref | 485.048 | 2 | <0.001 |
| Dosage^2^ |  |  | 6.32 (1.46) <0.001 |  |  |  |  |
| **GLU, mmol/L** |  |  |  |  |  |  |  |
| B1 (<5.8) |  |  |  |  |  |  |  |
| Dosage | 778 | 19.5(29.8) | 8,72 (3.80) 0.022 | 0.871 | 536.238 | 2 | <0.001 |
| Dosage^2^ |  |  | 5.96 (1.51) <0.001 |  |  |  |  |
| B2 (≥5.8） |  |  |  |  |  |  |  |
| Dosage | 787 | 20.0 (33.2) | 11.22 (4.61) 0.015 | ref | 355.123 | 2 | <0.001 |
| Dosage^2^ |  |  | 5.05 (1.83) 0.006 |  |  |  |  |
| **HDL, mmol/L** |  |  |  |  |  |  |  |
| B1 (<1.7) |  |  |  |  |  |  |  |
| Dosage | 779 | 18.0 (28.8) | 7.73(3.92) 0.049 | 0.097 | 416.527 | 2 | <0.001 |
| Dosage^2^ |  |  | 5.46 (1.55) <0.001 |  |  |  |  |
| B2 (≥1.7） |  |  |  |  |  |  |  |
| Dosage | 786 | 21.5 (34.0) | 11.84 (4.41) 0.007 | ref | 445.642 | 2 | <0.001 |
| Dosage^2^ |  |  | 5.60 (1.76) 0.001 |  |  |  |  |
| **TC, mmol/L** |  |  |  |  |  |  |  |
| B1 (<5.3) |  |  |  |  |  |  |  |
| Dosage | 779 | 19.3 (32.6) | 9.90 (4.52) 0.029 | 0.879 | 389.276 | 2 | <0.001 |
| Dosage^2^ |  |  | 5.52 (1.78) 0.002 |  |  |  |  |
| B2 (≥5.3） |  |  |  |  |  |  |  |
| Dosage | 786 | 20.2 (30.5) | 10.26 (3.91) 0.009 | ref | 483.984 | 2 | <0.001 |
| Dosage^2^ |  |  | 5.35 (1.56) <0.001 |  |  |  |  |
| **TG, mmol/L** |  |  |  |  |  |  |  |
| B1 (<1.4) |  |  |  |  |  |  |  |
| Dosage | 775 | 20.7 (33.7) | 10.30 (4.44) 0.021 | 0.048 | 447.183 | 2 | <0.001 |
| Dosage^2^ |  |  | 6.13 (1.76) <0.001 |  |  |  |  |
| B2 (≥1.4） |  |  |  |  |  |  |  |
| Dosage | 790 | 18.9 (29.3) | 10.51 (3.91) 0.007 | ref | 428.965 | 2 | <0.001 |
| Dosage^2^ |  |  | 4.52 (1.55) 0.004 |  |  |  |  |

^a^ Adjusted for center, sex, age, BMI, smoking and drinking status, C677T, baseline eGFR, baseline GLU, baseline HD-CL, baseline TC, baseline TG, SBP, and DBP.
